# *Bacillus cereus*–Attributable Primary Cutaneous Anthrax-Like Infection in Newborn Infants, India

**DOI:** 10.3201/eid2507.181493

**Published:** 2019-07

**Authors:** Lahari Saikia, Navonil Gogoi, Partha Pratim Das, Arunjyoti Sarmah, Kumari Punam, Bipanchi Mahanta, Simi Bora, Reeta Bora

**Affiliations:** Assam Medical College & Hospital, Dibrugarh, India

**Keywords:** Bacillus cereus, cutaneous, nosocomial, toxins, anthrax-like infections, infants, bacteria, India

## Abstract

During March 13–June 23, 2018, anthrax-like cutaneous lesions attributed to the *Bacillus cereus* group of organisms developed in 12 newborns in India. We traced the source of infection to the healthcare kits used for newborn care. We used multilocus sequence typing to characterize the 19 selected strains from various sources in hospital settings, including the healthcare kits. This analysis revealed the existence of a genetically diverse population comprising mostly new sequence types. Phylogenetic analysis clustered most strains into the previously defined clade I, composed primarily of pathogenic bacilli. We suggest that the synergistic interaction of nonhemolytic enterotoxin and sphingomyelinase might have a role in the development of cutaneous lesions. The infection was controlled by removing the healthcare kits and by implementing an ideal housekeeping program. All the newborns recovered after treatment with ciprofloxacin and amikacin.

The *Bacillus cereus* group includes ecologically diverse gram-positive and endospore-forming bacilli that are ubiquitous in the environment. The prominent members of this group are *B. wiedmanii*, *B. anthracis*, *B. cereus* sensu lato, *B. cereus* sensu stricto, *B. thuringiensis*, *B. weihenstephanensis*, *B. mycoides*, *B. pseudomycoides*, *B. cytotoxicus*, and *B. toyonensis*. Because the endospores of these species can resist extreme environmental conditions and thermal treatments, they are difficult to eliminate from processing chains of healthcare products and from clinical settings (*1*). The pathogenic potential of the *B. cereus* group varies from strains used as probiotics in animal feed to lethal and highly toxic strains (*2*,*3*). Thus, determining the degree to which pathogenic strains can be distinguished from nonpathogenic strains is essential.

*B. cereus* is well-known as a foodborne pathogen. In recent years, this bacterium was reported to cause several systemic and local nongastrointestinal infections in immunocompromised and immunocompetent persons (*4**,**5*). Specific populations, including intravenous drug abusers and patients with postsurgical or posttraumatic wounds, are at risk for these infections (*6**,**7*). In addition, numerous cases of fulminant infections similar to anthrax have been reported in healthy persons (*8**,**9*). Skin lesions of *B. anthracis* infection begin with a papule, which eventually becomes serosanguinous and develops a black eschar similar to some of the *B. cereus* skin lesions described by Henrickson et al. (*10*). Infections caused by *B. cereus* in newborns have been reported occasionally (*11**,**12*). We describe a cluster of 12 cases of severe anthrax-like cutaneous infections in otherwise healthy newborns attributed to the *B. cereus* group.

## Materials and Methods

### Case Study and Investigation

**Figure 1:**
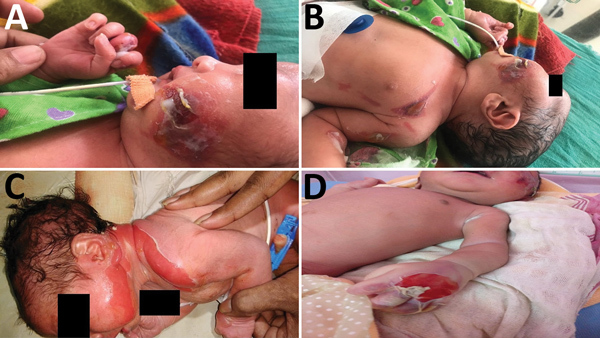
Newborn infants with cutaneous lesions mostly on face (A), left upper chest (B), neck (C), and hand (D), Assam Medical College & Hospital, Dibrugarh, India, 2018. This outbreak was later determined to have been caused by *Bacillus cereus*.

The Assam Medical College & Hospital (AMCH) is a tertiary care hospital in Dibrugarh, Assam, in northeasernt India. During March 13–June 23, 2018, extensive cutaneous vesicles or bullous lesions, mostly on the face, neck, and arm, developed in 12 newborns (8 boys, 4 girls); gas gangrene–like lesions eventually developed in 2 of the infants (Figure 1). All had been born healthy. All 12 newborns had a positive indication of sepsis. The initial clinical diagnosis was early-onset sepsis with staphylococcal scalded skin syndrome. Retrospectively, when records of these cases were analyzed, blood cultures were sterile or had growth of coagulase-negative *Staphylococcus aureus*.

This investigation focused on the labor room and the attached baby room because skin lesions developed within a few hours after delivery. Samples from exposed healthcare products, including the healthcare kits that contained items used during delivery, were cultured in nutrient broth and incubated at 37°C for 24 h. Upon confirmation of visible growth in nutrient broth, the samples were subcultured in blood and nutrient agar and incubated at 37°C for 24 h. Hand swab samples from hospital staff and the environment were cultured both aerobically and anaerobically. We obtained samples from the skin, armpit, and umbilical cord stump of newborns just after delivery at 2-day intervals. All samples from infants, staff, and the environment showed substantial growth of *Bacillus* species. All the bacteriology work was conducted in a Biosafety Level 3 laboratory. The institutional ethics committee (human) of AMCH approved this study.

### Intervention

On May 19, 2018, after confirmation of *B. cereus* group in the healthcare kits, the infection control officer from the AMCH Department of Microbiology advised using these kits only after they were autoclaved in a validated steam autoclave and terminal cleaning (i.e., extensive cleaning of all detachable objects in the room, cleaning of air duct surfaces in the ceiling, and thorough cleaning of everything downward to the floor) of the labor and the attached baby room was performed. However, on June 23, the same type of lesion developed in another newborn. When *B. cereus* outbreaks occur, obtaining control is difficult because these bacilli can survive long periods in the environment and are resistant to many commonly used sanitizing agents (*13*). After the June 23 case, an extensive terminal cleaning of the unit was done, along with staff training on appropriate housekeeping practices. All the instruments and containers were autoclaved, and surfaces were cleaned in 2 steps: first, with alkaline detergents, then with a disinfectant (D-125, Microgen, http://microgenindia.co). Beds were manually cleaned with detergent and water followed by heat treatment. Rooms were fogged with a sporicidal disinfectant containing hydrogen peroxide and silver nitrate (ECOSHIELD; Johnson & Johnson, https://www.jnj.com). All the healthcare kits were removed, and staff were advised to discontinue their use. Since June 23, 2018, no additional cases have been reported. 

### Identification of *B. cereus* Group

Based on the colony characteristics, β-hemolysis in blood agar, motility, production of lecithinase in egg yolk media, inability to ferment mannitol, and penicillin resistance, the isolates were designated as *B. cereus*. We subjected 4 representative strains to sequencing using the universal primer PF (5′-AGAGTTTGATCATGGCTCAG-3′) and PR (5′-GGACTACCAGGGTATCTAAT-3′) for the 16s rRNA gene (*14*).

### PCR Detection of Toxin-Encoding Genes

We performed PCR detection for toxins (*cytK*, *nheA*, *nheB*, *nheC*, *hblA*, *hblC*, *hblD*, *entFM*, *pi*-*plc*, and *sph*) and plasmid-encoding *B. anthracis* virulence factors (*cap*, *lef pag*, and *cya*) encoding genes. The primer sequences used for PCR are listed in the Appendix Table.

### Multilocus Sequence Typing Data Analysis

We selected 19 strains for molecular characterization. We used the *B. cereus* multilocus sequence typing (MLST) website (https://pubmlst.org/bcereus) that contains the partial sequences of the 7 housekeeping genes (*glp*, *gmk*, *ilv*, *pta*, *pur*, *pyc*, and *tpi*) (*15*). We conducted Sanger sequencing using Genetic Analyzer 3500 (Applied Biosystems, https://www.thermofisher.com). We compared the allele sequences with those available in the MLST database for assignment of allele numbers and sequence type (ST). We submitted all new alleles, MLST profiles (STs), and isolates to the MLST database. We obtained the population snapshot of the 1,795 STs available in the MLST database using goeBURST implemented in Phyloviz 2.0 using the default single-locus variant level (sharing at least 6/7 alleles) (*16*). The goeBURST Full MST (minimum spanning tree; http://www.phyloviz.net/goeburst) was done to identify BURST groups (BGs) among the STs identified in this study.

### Phylogenetic Analysis

The concatenated MLST sequences available at the MLST database were used for constructing maximum-likelihood trees. We used RAxML version 8 (*17*) implemented in RDP4 version 4.66 (*18*) with the GTRCAT model and a bootstrap resampling of 1,000 replicates.

### Diversity and Recombination Analysis

We calculated the length of each MLST locus, number of alleles, average nucleotide diversity (π), and number of polymorphic sites using DnaSP version 6.11.01 (*19*) based on the allelic sequences of the STs. We calculated the ratio of nonsynonymous to synonymous substitutions (dN/dS) to determine the selective pressure at each locus using the Nei and Gojobori method in START2 (*20*). The parameters dN and dS indicated average nonsynonymous and synonymous substitutions per site, respectively.

We conducted phylogenetic network analysis using Splits Tree version 4 (*21*) to identify lineages and recombination events within and across the lineages. We constructed the Splits Tree networks based on the concatenated sequences of STs using the neighbor-net algorithm with bootstrap resampling of 1,000 replicates. The resulting networks were analyzed using pairwise homoplasy index (PHI) test implemented in Splits Tree. A p value <0.05 indicated significant evidence of recombination.

We evaluated the linkage disequilibrium for the allelic data using LIAN version 3.7 (http://guanine.evolbio.mpg.de/cgi-bin/lian/lian.cgi.pl/query) (*22*). The standardized index of association (*I*_A_^S^) used for estimating linkage disequilibrium between alleles of the 7 MLST loci was calculated using the Monte Carlo method with 10,000 burn-in iterations. The *I*_A_^S^ values >0 and p<0.05 indicated significant linkage disequilibrium.

## Results

### Population Structure and BURST Group

MLST identified 14 STs among the selected 19 strains, including 5 predefined STs (ST75, ST127, ST266, ST380, and ST1465) and 9 new STs (ST1659, ST1660, ST1661, ST1662, ST1663, ST1664, ST1665, ST1667 and ST1668). Most (64.3%) identified STs were new.

**Figure 2:**
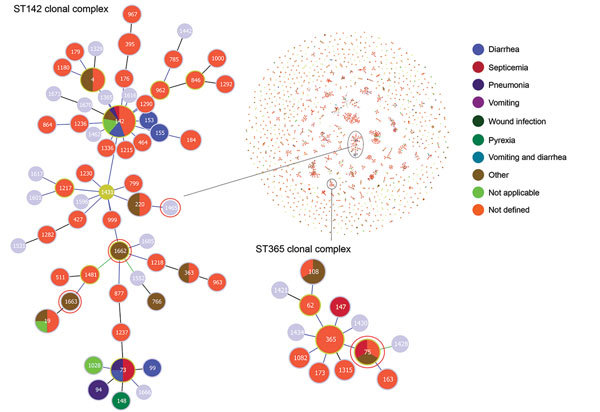
Population snapshot obtained using goeBURST (http://www.phyloviz.net/goeburst) of the 1,795 STs available to date in the *Bacillus cereus* multilocus sequence typing database overlaid by isolate data of human diseases. Each circle represents an ST. Size of the circle is logarithmically proportionate to the number of isolates represented by a given ST. Two ST clonal complexes are enlarged; STs highlighted in red circles were identified during investigation of an outbreak at Assam Medical College & Hospital, Dibrugarh, India, 2018. ST, sequence type.

**Figure 3:**
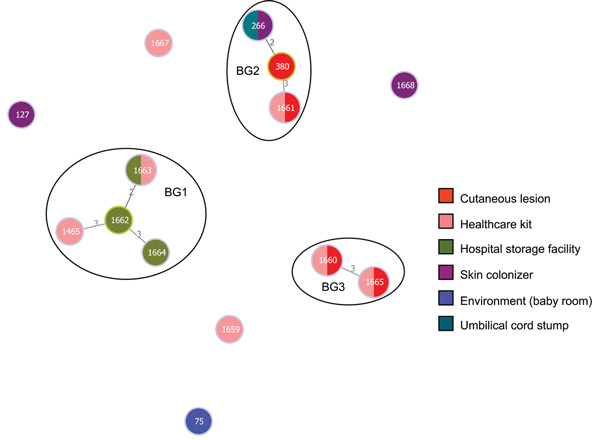
goeBURST full MST analysis (http://www.phyloviz.net/goeburst) at triple-locus variant level illustrating the evolutionary relationship within the 3 BGs identified during investigation of an outbreak at Assam Medical College & Hospital, Dibrugarh, India, 2018. Each circle represents a sequence type (ST), and its size is logarithmically proportionate to the number of isolates represented by that ST. The numbers over the line connecting 2 STs indicate the number of allele difference between them. The colors of the circles indicate the source of isolation. BG, BURST group.

The *B. cereus* MLST database clusters the available 1,795 STs into 10 major clonal complexes (CCs); a CC is a group of STs defined by goeBURST using the stringent group definition of single-locus variant level. The population snapshot obtained using goeBURST identified 3 STs (ST1465, ST1662, and ST1663) from the ST142 CC and 1 ST (ST75) from ST365 CC among the 14 STs identified in this study (Figure 2). The remaining STs were not a part of these major CCs. With a less stringent group definition of double-locus variant (DLV, sharing at least 5/7 alleles) or triple-locus variant (TLV, sharing at least 4/7 alleles) level, all of the STs in a goeBURST group cannot be considered as members of a single CC and hence referred to as BGs in our study. The goeBURST full MST analysis using the 14 STs from this study identified 3 BGs at TLV level (sharing at least 4/7 alleles) (Figure 3). The BG1 with ST1465, ST1662, ST1664, and ST1665 comprised strains from storage facilities and healthcare kits with ST1662 assigned as the putative primary founder. The BG2 included STs 266, 380, and 1661 with ST380 assigned as the putative primary founder. The BG2 consisted of strains from skin colonizers, umbilical cord stump, healthcare kits, and cutaneous lesions. The doubleton BG3 consisted of ST1660 and ST1665 with isolates recovered from healthcare kits and cutaneous lesions. We identified 5 singletons (not linked to any other ST): ST75, ST127, ST1659, ST1662, and ST1668.

### Phylogenetic Origin of the Strains

**Figure 4:**
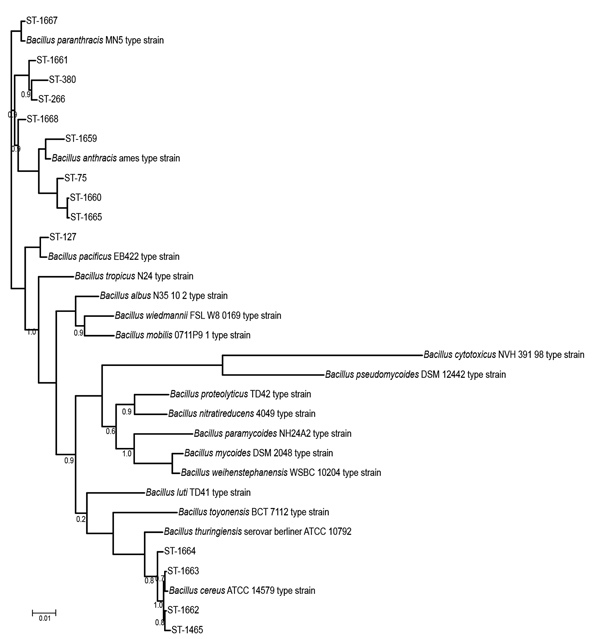
Phylogenetic relatedness of the 14 STs identified during investigation of an outbreak at Assam Medical College & Hospital, Dibrugarh, India, 2018, with 18 *Bacillus cereus* group species type strains. Scale bar indicates nucleotide substitutions per site.

**Figure 5:**
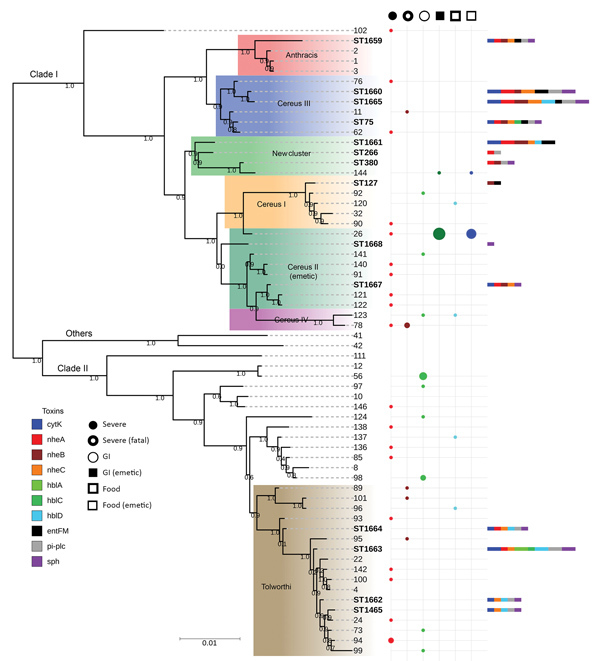
Maximum-likelihood tree constructed on the basis of concatenated sequences of the 51 *Bacillus cereus* STs illustrating the phylogenetic relatedness of the 14 STs identified during investigation of an outbreak at Assam Medical College & Hospital, Dibrugarh, India, 2018, and the 37 STs representing clinical isolates. The bootstrap support values for the nodes are indicated in decimals. The STs represented in bold letters are identified in this study. The color gradient boxes represent the various lineages found within the clades. The colored circle plot represents the number of isolates from various human diseases assigned to a particular ST, and each color represents a different human disease. The size of each circle is proportionate to the number of isolates. The multicolored bar indicates the number of toxin-encoding genes found in isolates represented by an ST and each color represents different toxin-encoding genes. The length of each colored bar is proportionate to the number of isolates positive for that toxin-encoding gene. GI, gastrointestinal; ST, sequence type. Scale bar indicates nucleotide substitutions per site.

The taxonomic identification of the 14 STs found in this study was done by constructing a phylogenetic tree with 18 *B. cereus* group species type strains (Figure 4). To illustrate the virulence potential of the strains, we constructed a phylogenetic tree (Figure 5) using the 14 STs from this study along with 38 STs representing 55 virulent isolates of *B. cereus* based on the selection made by Hoffmaster et al. (*23*). The STs clustered into 3 phylogenetic clades and were named to be consistent with previously defined phylogenetic clades by Priest et al. (*24*). Among the 14 STs we identified, 10 STs representing 14 strains were assigned to clade I, which comprised primarily pathogenic bacilli and mostly represented strains from healthcare kit and cutaneous lesion (*24*). Out of the 10 STs, 3 STs clustered into the cereus III/clade I lineage, 3 STs into a new cluster/clade I represented by ST144, 1 ST in cereus I/clade I lineage, and 2 STs in cereus II (emetic)/clade I lineage. None of the clade I–designated 10 STs grouped into the cereus IV lineage. Clade II consisted of 4 STs representing 5 strains grouped in tolworthi/clade II lineage and were representing strains mostly from the environment. None of the 14 STs identified in this study clustered in clade III (others).

### Distribution of Enterotoxins, Sphingomyelinase, and Phosphatidylinositol Phospholipase C Encoding Genes

The *cytK*, *sph*, and *pi-plc* genes encoding cytotoxin K, sphingomyelinase (SMase), and phosphatidylinositol phospholipase C (Pi-Plc), respectively, were commonly detected in 73.7% (n = 14) of the 19 selected strains, followed by *nheA* and *nheC* in 68.4% (n = 13), *nheB* in 52.6% (n = 10), and *entFM* in 42.1% (n = 8) (Table 1). Of the 14 strains previously designated to clade I, the *nheABC* gene complex (*nheA*, *nheB*, and* nheC*) encoding the nonhemolytic enterotoxin (Nhe) was detected in 8 strains and *entFM* gene encoding enterotoxin FM was also detected in 8 strains. None of the clade II assigned strains were detected positive for the *nheABC* gene complex and the *entFM* gene. The *hbl* gene complex (*hblA*, *hblC*, and *hblD*) encoding hemolysin BL (HBL) was found in only 1 strain designated to clade II, whereas none of the clade I–assigned strains harbored this complex (Table 2). Both *hblA* and *hblC* were detected in 1 each of the 19 strains. None of the strains were positive for genes encoding *B. anthracis* plasmid-mediated virulence factors (Table 1).

**Table 1:** Toxin-encoding gene profile of 19 selected *Bacillus cereus* strains, Assam Medical College & Hospital, Dibrugarh, Assam, , India, 2018*

Strain	Source	ST	Clade	*cytK*	*nheABC*				*hblCDA*			*entFM*	*pi-plc*	*sph*
					*nheA*	*nheB*	*nheC*		*hblA*	*hblC*	*hblD*			
AMCER1	Healthcare kit	1659	I	+	+	+	+		–	–	–	+	+	+
AMCER2	Healthcare kit	1660	I	+	+	–	+		–	–	–	+	+	+
AMCER3	Cutaneous lesion	1660	I	+	+	+	+		–	–	–	+	+	+
AMCER4	Healthcare kit	1661	I	+	+	+	+		–	–	+	+	–	–
AMCER5	Cutaneous lesion	1661	I	+	+	+	–		–	–	–	+	–	–
AMCER6	Healthcare kit	1465	II	+	–	–	+		–	–	+	–	+	+
AMCER7	Healthcare kit	1667	I	+	+	+	+		–	–	–	–	–	+
AMCER8	Hospital storage facility	1662	II	+	–	–	+		–	–	+	–	+	+
AMCER9	Hospital storage facility	1663	II	+	+	–	+		+	–	+	–	+	+
AMCER11	Healthcare kit	1663	II	+	–	–	–		+	+	+	–	+	+
AMCER10	Hospital storage facility	1664	II	+	+	–	+		–	–	+	–	+	+
AMCER12	Healthcare kit	1665	I	+	+	+	+		–	–	+	+	+	+
AMCER15	Cutaneous lesion	1665	I	+	+	+	+		–	–	+	–	+	+
AMCER13	Umbilical cord stump	266	I	–	–	–	–		–	–	–	–	+	–
AMCER14	Environment	75	I	+	+	+	+		–	+	–	+	+	+
AMCER18	Skin colonizer	266	I	–	+	–	–		–	–	–	–	+	–
AMCER19	Cutaneous lesion	380	I	–	+	+	+		–	–	–	–	+	+
AMCER20	Skin colonizer	1668	I	–	–	–	–		–	–	–	–	–	+
AMCER21	Skin colonizer	127	I	–	–	+	–		–	–	–	+	–	–

*ST, sequence type; +, positive; –, negative.

**Table 2:** Interclade variability of *Bacillus cereus* toxin–encoding genes and gene complexes, Assam Medical College & Hospital, Dibrugarh, Assam, India, 2018

PCR characterization	Phylogenetic group, %*	
	Clade I	Clade II
*pi-plc*	9 (64.3)	5 (100)
*sph*	9 (64.3)	5 (100)
*cytK*	9 (64.3)	5 (100)
*entFM*	8 (57.1)	0
*nheABC*	8 (57.1)	0
*hblCDA*	0	1 (20)
Total.	14	5

*The percentage of each cell corresponds to the number of strains within a given clade positive for the encoding gene. Strains were classified as positive for a gene complex if all the genes in a given complex were detected by PCR.

### Sequence and Allelic Diversity

Sequence alignment of each of the 7 MLST loci showed no insertion or deletion with sizes ranging from 348 bp (*pur*) to 504 bp (*gmk*). The number of alleles at each locus ranged from 4 (*gmk*) to 11 (*ilv* and *tpi*). The dN/dS values indicate selective pressure on protein-coding genes; dN/dS >1 indicates positive and dN/dS <1 negative selective pressure. The dN/dS ratio for each locus varied from 0.0076 (*ilv*) to 0.0547 (*pur*), indicating strong negative/purifying selective pressure on these genes (Table 3).

**Table 3:** Sequence and allelic diversity of the 7 multilocus sequence type loci of *Bacillus cereus*, Assam Medical College & Hospital, Dibrugarh, Assam, India, 2018

Locus	Size, bp	Guanine + cytosine content, %	Allele	Polymorphic site	π	dN/dS
*Glp*	372	38.3	10	18	0.01607	0.0312
*Gmk*	504	38.2	4	24	0.02546	0.0214
*Ilv*	393	45.1	11	58	0.06283	0.0076
*Pta*	414	40.5	8	21	0.02088	0.0077
*Pur*	348	38.5	9	42	0.05388	0.0098
*Pyc*	363	40.6	8	48	0.05460	0.0301
*Tpi*	435	44.1	11	18	0.01492	0.0547

### Recombination Analysis

**Figure 6:**
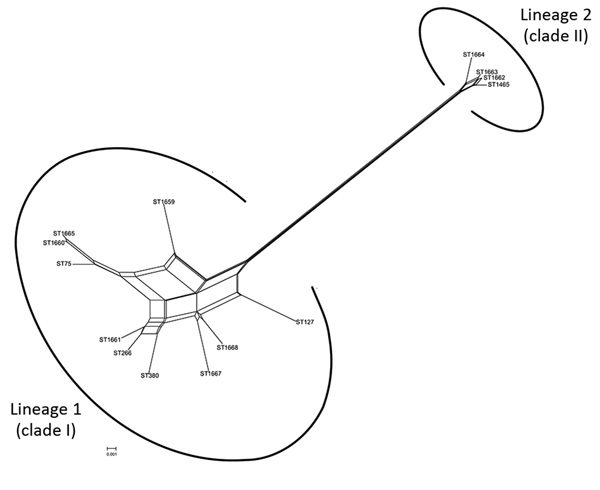
Phylogenetic network analysis using Splits Tree (*21*) identified 2 lineages among the whole population of 14 STs, Assam Medical College & Hospital, Dibrugarh, Assam, Northeast, India, 2018. ST, sequence type.

The Splits Tree network for the 14 STs representing the 19 selected strains identified 2 lineages among them (Figure 6). Lineage 1 comprised STs 75, 127, 266, 380, 1660, 1659, 1661, 1665, 1667, and 1668 previously designated to clade I. The STs 1465, 1662, 1663, and 1664 previously designated to clade II were in lineage 2. We observed extensive reticulations across the lineages and within lineage 1. The PHI test also provided significant evidence of recombination for the whole population (14 STs) and lineage 1 (p<0.05). However, the *I*_A_^S^ values, for estimation of linkage disequilibrium, differed significantly from 0 for the entire population, as well as for the lineages, indicating the existence of linkage disequilibrium between the loci or a clonal population structure (Table 4).

**Table 4:** Estimation of linkage disequilibrium of *Bacillus cereus*, Assam Medical College & Hospital, Dibrugarh, Assam, India, 2018

Group	Linkage disequilibrium	
	*I*_A_^S^	p value
Lineage 1	0.3800	<0.05
Lineage 2	0.3471	<0.05
Whole population	0.4573	<0.05

## Discussion

MLST data analysis identified a genetically diverse population of 14 STs representing the 19 selected *B. cereus* strains because most of the STs were identified as new. Population snapshot using goeBURST illustrated the rare occurrences of clinical cases among the *B. cereus* group. Among the 14 STs, 3 were from the ST142 CC and 1 from the ST365 CC, indicating evolutionary descent from worldwide clones. The goeBURST Full MST analysis of the 14 STs identified only 3 BGs even at the TLV level; the rest were identified as singletons, which again illustrates the high genetic diversity. Most of the strains isolated from healthcare kits and cutaneous lesions were represented by new STs, suggesting that greater diversity was possibly because of adaptation to the new niche.

The phylogenetic origin of the 19 strains was determined to investigate whether any of the 14 STs representing these strains clustered into the previously described clade I, composed primarily of pathogenic bacilli (*24*). The clade I–designated 10 STs were distributed into various lineages of clade I and were closely related to the Anthracis lineage, as described by Hoffmaster et al. (*23*). Among them, ST1659, a new ST representing a strain isolated from a healthcare kit, had the closest relationship to the Anthracis lineage and shared the same *gmk*, *pta*, *pur*, and *pyc* alleles with the Ames anthracis strain (ST1). The ST75 isolate from ST365 CC, which has been reported earlier for representing a severe septicemic *B. cereus* strain, shared the same *gmk* and *pta* alleles with the *B. anthracis* strains (*25*). 

Several studies have demonstrated that *B. cereus* isolates closely related to *B. anthracis* are of clinical rather than environmental origin (*26**,**27*).‬‬‬‬‬‬‬‬‬‬‬‬‬‬‬‬‬‬‬‬‬‬‬‬‬‬‬ All the clade I–designated strains were negative for genes encoding *B. anthracis* virulence factors. Alternatively, these factors might not be necessary for severe nongastrointestinal infections because isolates from severe cases have been reported to be negative for plasmids (*8*). Most of the clade I–assigned STs represented strains recovered from healthcare kits, suggesting these strains might be responsible for cutaneous lesions. Three clade II–assigned STs were from ST142 CC, comprising mostly foodborne isolates with potential to cause foodborne illness (*28*). Hence, the potential role of the strains represented by these 3 STs in the development of cutaneous lesions is arguable. 

Phylogenetic network analysis using Splits Tree identified 2 lineages among the 14 STs identified in this study. All the clade I–designated STs clustered in lineage 1, whereas all the clade II–designated STs clustered in lineage 2. Extensive reticulations in Splits Tree networks and PHI test provided significant evidence of recombination across the lineages and within lineage 2. Even though the *I*_A_^S^ and dN/dS values predicted an overall clonal population structure, the high genetic diversity and recombination might have enabled the population to enhance fitness and survive.‬ ‬‬‬‬‬

To evaluate the interclade variability of toxin-encoding genes, we performed PCR detection of these genes. The *nheABC* gene complex and *entFM* gene were detected only among the clade I–designated strains, whereas none of the clade II–identified strains were positive for the *nheABC* gene complex and the *entFM* gene. The Nhe enterotoxin is considered to be the major virulence factor in *B. cereus* diarrheal disease (*29*). The synergistic interaction of Nhe and SMase in in vitro cytotoxicity has been demonstrated (*30*). All the members of the *nheABC* gene complex are required to form functional transmembrane pores for the entry of SMase into the epithelial cell membrane, otherwise inaccessible, and could result in cell membrane destabilization, as well as cell apoptosis through the ceramide intracellular signaling pathway (*31**,**32*). This case might be valid in our study because the clade I–designated strains harbored the *nheABC* gene complex, as well as the *sph* gene. Several findings have suggested that enterotoxin FM might be a potential cell wall peptidase involved in mutant bacterial shape, impairment in motility, and adhesion to eukaryotic cells and thus might be responsible for the virulence of the clade I–assigned strains because most of them harbored this gene (*33*). The *hblA* gene encoding the binding subunit C component and, *hblC* gene encoding the L_2_ lytic component sparsely detected among the 19 strains. The tripartite HBL enterotoxin requires all its components for maximum enterotoxic activity (*34*). Moreover, the *cytK* and *hbl* enterotoxin genes are often absent in *B. cereus* strains isolated from disease outbreaks, which argues against its potential role to elicit disease (*35**,**36*). 

In this investigation, only 12 newborns were infected, even though the kits also were used for other newborns. Thus, development of nongastrointestinal infections in newborns is complex and might depend on factors such as the number of spores exposed, the presence of a virulent and avirulent cluster of microorganisms, toxin expression and interaction, and host conditions. Moreover, seasonal variation of increased count and germination of *B. cereus* spores in spring and summer have been reported (*37**–**39*). In this outbreak, lesions occurred during April in 6 newborns, May in 4, and March and June in 1 each. However, a thorough investigation is needed to understand the complexity of these infections. Our findings, along with previous reports, reinforce the idea that the members of the *B. cereus* group are underestimated emerging pathogens that can be involved in fatal nosocomial infections.

The cutaneous infections attributed to the *B. cereus* group in most of the cases in this study occurred in the exposed areas of the skin because they are often in contact with the environment and are prone to microscopic skin abrasions (*39*). The spores from the healthcare kits might have invaded the skin of newborns through these microscopic skin abrasions formed during baby cleaning (*39*). Moreover, vernix caseosa, a waxy substance covering the skin of newborns, often requires cleaning and might also be the cause of microscopic skin abrasions. Once in contact with skin, spores germinate and enterotoxin production occurs (*39*). In addition, toxicoinfection can occur because the kits contain all the items required to conduct labor, including contaminated gloves. Among the infants in this report, duration of labor ranged from 6 to 10 hours, so germination and toxin production might have occurred in the birth canal and accounted for the initial lesions that later extended from contact with the cleaning linens inside the kit and led to additional spore germination and toxin production.

Clinically, the lesions started as bullous or ruptured bullous lesions with extensive and rapidly spreading cellulitis. Two lesions eventually developed into gas gangrene–like infections as reported previously (*4**,**40*). However, in all 12 cases, blood culture was negative for *B. cereus*. Henrickson et al. reported the primary cutaneous infections caused by *B. cereus* in the absence of positive blood cultures (*10*,*39*). Extensive soft tissue involvement with gas gangrene infections in the first few newborns might have resulted from the initial use of β-lactam antimicrobial drugs because the existence of β-lactamase in sporulated *Bacillus* species has been predicted (*41*). All newborns recovered after treatment with ciprofloxacin and amikacin.

In conclusion, *B. cereus* primary cutaneous infection in newborns without bacteremia can occur from contaminated environments in hospitals. Bullous lesions or cellulitis during or just after delivery should be included in the differential diagnosis, and caution should be taken in initiating β-lactam antimicrobial drug treatment.

Appendix Primer sequences used in study of *Bacillus cereus*–attributable primary cutaneous anthrax-like infection in newborn infants, India.
