# Supplementary material for: Bacillus cereus–Attributable Primary Cutaneous Anthrax-Like Infection in Newborn Infants, India
Source: Emerg Infect Dis. 2019 Jul;25(7):1261–70. doi: 10.3201/eid2507.181493 (PMC6590766; doi:10.3201/eid2507.181493)
Supplement: Appendix — Primer sequences used in study of Bacillus cereus–attributable primary cutaneous anthrax-like infection in newborn infants, India. [file 18-1493-Techapp-s1.pdf]

Article DOI: https://doi.org/10.3201/eid2507.181493

Bacillus cereus–Attributable Primary Cutaneous Anthrax-Like Infection in Newborn Infants, India

Appendix

Appendix Table. Primer sequences used in this study*

Target gene	Sequence (5′–3′)	Amplicon size, bp	Annealing temperature, °C	Reference/GenBank accession no.
cytK	F: CAACCCAGTTTGCAGTTCCG<br>R: GGCGCTAGTGCAACATTACG	601	55	AJ277962
nheA	F: GTTAGGATCACAATCACCGC<br>R: ACGAATGTAATTTGAGTCGC	755	48	(*1*)
nheB	F: TTTAGTAGTGGATCTGTACGC<br>R: TTAATGTTCGTTAATCCTGC	743	45	(*1*)
nheC	F: TGGATTCCAAGATGTAACG<br>R: ATTACGACTTCTGCTTGTGC	683	47	(*1*)
entFM	F: AAAGAAATTAATGGACAAACTCAAACTCA<br>R: GTATGTAGCTGGGCCTGTACGT	596	50	(*2*)
hblC	F: GATACTCAATGTGGCAACTGC<br>R: TTGAGACTGCTCGTCTAGTTG	740	52	(*3*)
hblD	F: ACCGGTAACACTATTCATGC<br>R: GAGTCCATATGCTTAGATGC	829	48	(*1*)
hblA	F: AAGCAATGGAATACAATGGG<br>R: AGAATCTAAATCATGCCACTGC	1154	48	(*1*)
sph	F: CGTGCCGATTTAATTGGGGC<br>R: CAATGTTTTAAACATGGATGCG	558	48	(*4*)
pi-plc	F: CGCTATCAATGGACCATGG<br>R: GGACTATTCCATGCTGTACC	569	51	(*5*)
cya	F: GCGTTTTCTTTAGCGTTTTC<br>R: CTACATTTTTAGCCATCACTTCT	727	48	(*6*)
cap	F: CTGAGCCATTAATCGATATG<br>R: TCCCACTTACGTAATCTGAG	846	55	(*7*)
pag	F: TCCTAACACTAACGAAGTCG<br>R: GAGGTAGAAGGATATACGGT	596	55	(*8*)
lef	F: TGAACCCGTACTTGTAATCCAATC<br>R: ATCGCTCCAGTGTTGATAGTGCT	475	53	(*9*)

*F, forward; R, reverse.

References

1. Guinebretière MH, Broussolle V, Nguyen-The C. Enterotoxigenic profiles of food-poisoning and food-borne *Bacillus cereus* strains. J Clin Microbiol. 2002;40:3053–6. PubMed http://dx.doi.org/10.1128/JCM.40.8.3053-3056.2002

2. Kim J-B, Kim J-M, Cho S-H, Oh H-S, Choi NJ, Oh D-H. Toxin genes profiles and toxin production ability of *Bacillus cereus* isolated from clinical and food samples. J Food Sci. 2011;76:T25–9. PubMed http://dx.doi.org/10.1111/j.1750-3841.2010.01958.x

3. Chon JW, Kim JH, Lee SJ, Hyeon JY, Song KY, Park C, et al. Prevalence, phenotypic traits and molecular characterization of emetic toxin-producing *Bacillus cereus* strains isolated from human stools in Korea. J Appl Microbiol. 2012;112:1042–9. PubMed http://dx.doi.org/10.1111/j.1365-2672.2012.05277.x

4. Hsieh YM, Sheu SJ, Chen YL, Tsen HY. Enterotoxigenic profiles and polymerase chain reaction detection of *Bacillus cereus* group cells and B. cereus strains from foods and food-borne outbreaks. J Appl Microbiol. 1999;87:481–90. PubMed http://dx.doi.org/10.1046/j.1365-2672.1999.00837.x

5. Hansen BM, Damgaard PH, Eilenberg J, Pedersen JC. Molecular and phenotypic characterization of *Bacillus thuringiensis* isolated from leaves and insects. J Invertebr Pathol. 1998;71:106–14. PubMed http://dx.doi.org/10.1006/jipa.1997.4712

6. Shangkuan YH, Chang YH, Yang JF, Lin HC, Shaio MF. Molecular characterization of *Bacillus anthracis* using multiplex PCR, ERIC-PCR and RAPD. Lett Appl Microbiol. 2001;32:139–45. PubMed http://dx.doi.org/10.1046/j.1472-765x.2001.00881.x

7. Hutson RA, Duggleby CJ, Lowe JR, Manchee RJ, Turnbull PC. The development and assessment of DNA and oligonucleotide probes for the specific detection of *Bacillus anthracis*. J Appl Bacteriol. 1993;75:463–72. PubMed http://dx.doi.org/10.1111/j.1365-2672.1993.tb02803.x

8. Beyer W, Glöckner P, Otto J, Böhm R. A nested PCR method for the detection of *Bacillus anthracis* in environmental samples collected from former tannery sites. Microbiol Res. 1995;150:179–86. PubMed http://dx.doi.org/10.1016/S0944-5013(11)80054-6

9. Kim K, Seo J, Wheeler K, Park C, Kim D, Park S, et al. Rapid genotypic detection of *Bacillus anthracis* and the *Bacillus cereus* group by multiplex real-time PCR melting curve analysis. FEMS Immunol Med Microbiol. 2005;43:301–10. PubMed http://dx.doi.org/10.1016/j.femsim.2004.10.005
